# Vat Photopolymerization 3D Printing of Hydrogels with Re-Adjustable Swelling

**DOI:** 10.3390/gels9080600

**Published:** 2023-07-25

**Authors:** Pedro Liz-Basteiro, Felipe Reviriego, Enrique Martínez-Campos, Helmut Reinecke, Carlos Elvira, Juan Rodríguez-Hernández, Alberto Gallardo

**Affiliations:** 1 Instituto de Ciencia y Tecnología de Polímeros (ICTP), CSIC, C/Juan de la Cierva 3, 28006 Madrid, Spain; pedroliz@ictp.csic.es (P.L.-B.); freviriegop@ictp.csic.es (F.R.); e.martinez.campos@csic.es (E.M.-C.); hreinecke@ictp.csic.es (H.R.); celvira@ictp.csic.es (C.E.); 2 Grupo de Síntesis Orgánica y Bioevaluación, Instituto Pluridisciplinar (IP), UCM, Unidad Asociada al CSIC por el ICTP y el IQM, Paseo de Juan XXIII 1, 28040 Madrid, Spain

**Keywords:** 3D printing, vat polymerization, hydrogel, re-adjustable swelling, hydrolysable crosslinker, additive manufacturing, DLP, UV curing

## Abstract

Vat photopolymerization typically prints highly crosslinked networks. Printing hydrogels, which are also networks but with a high swelling capacity in water and therefore with low crosslinking density, is a challenge for this technique. However, it may be of interest in medicine and in other areas, since it would allow for the preparation of this type of 3D-shaped material. In this work, an approach for printing hydrogels via vat photopolymerization that uses a mixture of stable and hydrolysable crosslinkers has been evaluated so that an initial highly crosslinked network can be printed, although after hydrolysis it becomes a network with low crosslinking. This approach has been studied with PEO/PEG-related formulations, that is, with a PEG-dimethacrylate as a stable crosslinker, a PEO-related derivative carrying *β*-aminoesters as a degradable crosslinker, and PEG-methyl ether acrylate and hydroxyethyl acrylate as monofunctional monomers. A wide family of formulations has been studied, maintaining the weight percentage of the crosslinkers at 15%. Resins have been studied in terms of viscosity, and the printing process has been evaluated through the generation of Jacobs working curves. It has been shown that this approach allows for the printing of pieces of different shapes and sizes via vat photopolymerization, and that these pieces can re-ajust their water content in a tailored fashion through treatments in different media (PBS or pH 10 buffer).

## 1. Introduction

Vat photopolymerization 3D printing, which historically was the first additive manufacturing (AM) technology, has emerged as a revolutionary technique in this field, enabling the fabrication of intricate structures with high precision and accuracy [1]. Three-dimensional printing by means of vat photopolymerization commonly employs resins with high contents of di- or multifunctional polymerizable crosslinkers [2,3], thus yielding parts with a highly crosslinked network structure with dimensional stability and little or no swelling capacity. However, crosslinked networks capable of swelling in water, that is, hydrogels, are materials of great interest in biomedicine and in other applications [4,5,6,7]. The possibility of being able to prepare hydrogel-type objects by means of vat photopolymerization is very attractive for preparing systems with a customized 3D structure and with the high resolution characteristic of this 3D printing technology [8].

To print hydrogels, the crosslinking density should be designed, in principle, according to the characteristics of the targeted swelling. According to literature data, selected as an example, if it is desired to print an acrylate-based hydrogel that mimics soft tissues, that is, with a certain amount of water (for example, containing double the amount of water of a polymer), the photocurable formulation must contain around 1 wt. % of a crosslinker such as ethylene glycol dimethacrylate [9]. This percentage, which is much lower than those crosslinking densities obtained using standard resins, may not be compatible with certain aspects of vat photopolymerization 3D printing because the technique usually requires a minimum amount of crosslinkers to obtain parts with adequate resolution and desirable mechanical properties [3,10,11,12]. On certain occasions, low crosslinking is related to poor adhesion to the platform.

In order to overcome this issue, one strategy that can allow for the printing of hydrogel-type networks with the desired swelling capacity using vat photopolymerization 3D printing [13] involves the preparation of formulations containing a mixture of stable and labile crosslinkers. In this way, the crosslinker content in the resin would be enough for printing pieces with good definition and mechanical properties, although the printed part could be subsequently readjusted by selectively breaking the labile crosslinking bridges. As a result, the final network will have a crosslinking density defined by the amount of stable crosslinker. If the labile character refers to hydrolytic sensitivity, as is the case in this work, the printed network will be able to readjust in water, a non-toxic and ubiquitous solvent, rendering it very attractive for commercial implementation.

In addition, the network readjustment may be of interest for certain applications, such as filling cavities in regenerative medicine [14], the use of hydrogels as supports and reservoirs in hydroponics and aquaponics [15], or for pharmaceutical and cosmetic manufacturing [16,17], which could benefit from a controlled increase in size upon swelling in water.

When designing the printing of a network precursor of a hydrogel, it must be taken into account that the size of the fresh printed part is smaller than that of the swollen networks. If the swelling is isotropic [18,19], the swollen part replicates the shape of the printed part, and the original dimensions and their increase via swelling can be designed to obtain a final custom hydrogel.

Our group has recently described a related strategy to prepare sacrificial molds in water via vat photopolymerization [20]. In that work, resins containing hydrolysable structures as crosslinkers were prepared and evaluated so that complex molds could be printed with good resolution. The obtained molds were then successfully evaluated for the preparation of both thermoplastic screws via plastic melting, mold filling, cooling and sacrificing the mold in aqueous media, as well as silicone lattices prepared by filling the mold with precursors, chemical crosslinking, and again sacrificing the mold in aqueous media.

Based on this previous experience, in this work, different formulations containing mixtures of stable and hydrolysable crosslinkers have been optimized and evaluated for 3D printing. The resulting printed parts were characterized in terms of the size variation and water content. Liquid crystal display (LCD) 3D printing has been used in this work. LCD is today a cost-effective and widely employed technology for 3D printing using UV-curable photosensitive resins [21,22]. Moreover, the high resolution screens available today, combined with the possibility to create a complete layer in a short time, have made this technology one of the fastest and most precise 3D printing alternatives [23]. Although LCD 3D printing has been used in this work, the procedure studied here could be extended following slight modifications of the photosensitive resin composition to other vat photopolymerization techniques, such as stereolithography (SLA) and digital light processing (DLP).

## 2. Results and Discussion

### 2.1. Design of the Photopolymerizable Resin and Optimization of the 3D Printing Parameters

**Figure 1:**
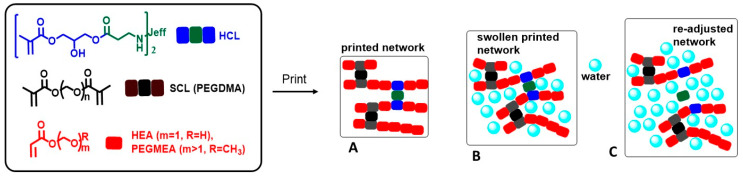
Scheme of the strategy used in this work for printing re-adjustable hydrogels via vat photopolymerization. (**A**) A 3D printed part obtained directly after printing and post-curing. (**B**) Initial swelling in PBS. (**C**) A 3D printed part upon swelling in basic media due to the HCL hydrolysis. The jeff tag refers to the spacer of the Jeffamine (R of H_2_N-R-NH_2_).

Figure 1 schematically shows the strategy used in this work as well as the structures of the components of the formulation. Three-dimensional printing via vat photopolymerization renders type A networks, which have a crosslinking density defined by the sum of the stable crosslinker, SCL, and the hydrolysable crosslinker, HCL. This A network, before any swelling readjusting, is capable of uptaking some amount of water to become a type B network. B networks are then capable of readjusting their swelling upon selective hydrolysis of the hydrolysable crosslinking agents to give rise to C, with network C being the network with the maximum readjustment in swelling (only the permanent crosslinking agents ensure the hydrogel’s integrity). Thus, the adjustable network design is based on the simultaneous use of the SCL and HCL. In this way, the adjustable character of hydrogels refers to the ability to modulate the degree of swelling, starting from an initial value (network B in the Figure 1), through the controlled hydrolysis of the HCL bridges. The final (and maximum) swelling is defined by the residual SCL bridges (after hydrolysis of all the HCL; network C in the Figure 1).

In addition to the crosslinkers, monofunctional monomers responsible for modulating certain properties of the final hydrogel were added to the formulation. Results from our previous work were used to select the components and the starting parameters [20]. A Jeffamine derivative carrying activated *β*-aminoesters for hydrolysis was chosen as the HCL, because it has previously shown its effectiveness in the preparation of sacrificial templates. PEG derivatives (PEGDMA-550 as the crosslinker and PEGMEA-480 as the monofunctional monomer) as well as their ethoxylated low molecular weight homologue (HEA) were chosen as structures of the SCL and monofunctional monomers in order to build PEO-related structures. In this sense, Jeffamine is also a PEO-related structure.

The crosslinking percentage was established as 15 wt. % (total amount of the SCL + HCL), as this value has been shown before to be the minimum required to print parts with good resolution and acceptable mechanical properties [20].

Initially, the influence of the monofunctional monomers (HEA and PEGMEA) on the printing process and the properties of the printed pieces has been addressed. For this purpose, the HCL/PEGDMA weight percentages were set at 7.5/7.5, and the samples were prepared by varying the HEA/PEGMEA weight ratio, as shown in Table 1. This series was labelled as PEGDMA_7.5_-HEA_y_, where *y* is the weight percentage of the HEA.

First of all, in order to evaluate the photopolymerizable resin printability, a rheological study was carried out to measure the viscosity as a function of the shear rate. All the samples have shown Newtonian behavior (see Figure S1 in SI). Although there is a certain dependence on the composition (the higher the amount of HEA, the lower the viscosity), the viscosities remain in all cases below 0.050 Pa·s, evidencing their suitability for DLP/LCD printing, which requires low viscosities [24,25]. The values of the shear rate measured for a frequency of 1 s^−1^ have been quoted in Table 1.

**Figure 2:**
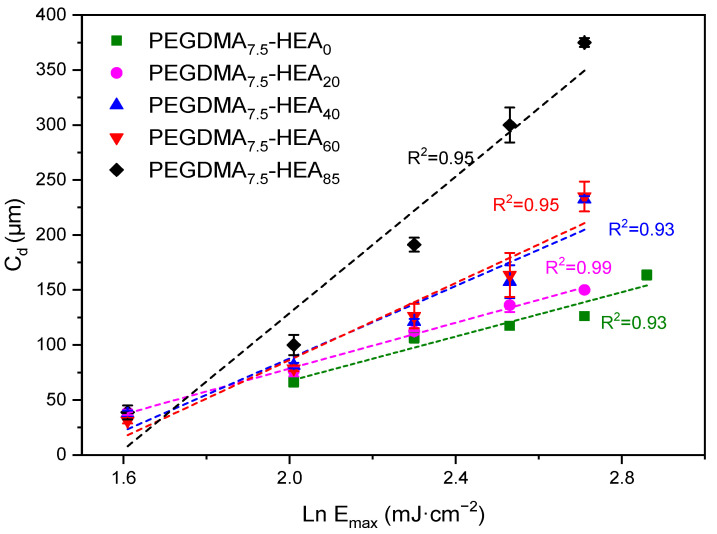
Working curves for the PEGDMA_7.5_-HEA_y_ series when exposed to 405 nm light (0.5 mW·cm^−2^). Data correspond to (from right to left in each group of data) exposure times of 35, 30, 25, 20, 15 and 10 s. Linear regressions are included.

In order to select the optimal printing parameters, and more specifically, the exposure time for a layer of 100 µm, the Jacobs working curves [26,27,28,29] (see Figure 2) were determined as described in the Experimental Section. These lines, which represent the cured depth (C_d_) against the energy that the printer lamp radiates on the surface of the part for different exposure times, allow for the selection of the most appropriate exposure times for the layer height selected in the printing parameters. As can be observed in Figure 2, for each exposure time, the C_d_ decreases as the amount of HEA decreases. This working line provides information about the E_c_ (mJ·cm^−2^), the starting point for the transition from liquid to solid, and the D_p_ (µm), the penetration depth. The parameters calculated from these curves are presented in Table 1.

The HEA/PEGMEA weight ratio does not have a great influence on the E_c_, although it does on the Cd. The greater the amount of HEA, the greater the D_p_ for each exposure time. Table 1 shows that the variation in the HEA/PEGMEA ratio has a great influence on the molar crosslinking density: the more HEA, the lower the molar crosslinking density, which means, in principle, that it will require longer irradiation times (or energy) to reach a gel structure. In addition, by varying the HEA/PEGMEA ratio, the ratio between the low molecular weight monomer (HEA) and the macromonomer (PEGMEA) is also being varied. It has been described that the degree of functionality of the components (mono- vs. difunctional) and their molecular weight have a profound influence on both the gel point and the curing depth [30].

Based on the graphs shown in Figure 2, and taking into account that the objective is to obtain a layer height of 100 microns, 20 s was chosen as the common exposure time for all the printings. For this exposure time, the different systems result in curing depths close to the target value.

To assess the printability and make an initial characterization of the dimensional readjustment, pieces with dimensions of 2.0 × 2.0 × 0.2 cm^3^ were printed for the different formulations. According to previous studies [31], the photoabsorber Sudan I was added at 1.35 wt. %, to limit the UV light penetration. Please note that Sudan I was already used in a previous study to obtain the Jacobs working curves. The optimized printing parameters employed to print the parts are summarized in the Experimental Section.

It is worth mentioning that an increase in the amount of HEA in the photopolymerizable mixture leads to more resistant and less brittle parts. This fact may be associated with the large variation in the crosslinking molar density, i.e., an increase in the HEA content involves a reduction in the crosslinking molar density.

### 2.2. 3D Printed Part Swelling: Toward a Selective Rupture of the Hydrolysable Crosslinking Agents

**Figure 3:**
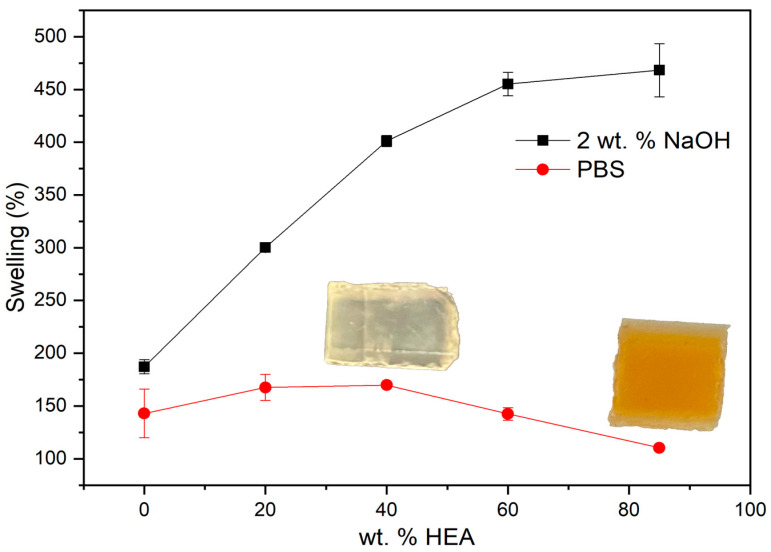
Red circles: swelling at equilibrium in PBS. Black squares: swelling after 24 h in water with 2 wt. % NaOH; in both cases, data are for samples of the PEGDMA_7.5_-HEA_y_ series.

The pieces were immersed either in PBS or in basic water (2 wt. % of NaOH) and gravimetrically monitored to determine their water content and calculate the corresponding swelling according to Equation (1). In PBS, a gradual increase in swelling was observed until reaching an equilibrium value varying in the 100–170% swelling range, depending on the composition. This equilibrium value, which is reached between 24 and 96 h (depending on the system), is plotted in Figure 3. Interestingly, although the crosslinking density decreases with an increasing HEA content, this does not translate into a correlative increase in swelling. Swelling increases slightly up to 40 wt. % HEA but then decreases, remaining in all cases above 100%. To the naked eye, it is observed that very HEA-rich hydrogels have a transparent outer layer and a colored core, which is probably slightly swollen. This behavior can be as explained follows. With an increasing HEA content, on the one hand, there is initially a slight increase in swelling in PBS due to the significant decrease in the molar percent crosslinking. From 40%, on the other hand, these variations in the degree of crosslinking are not as high, and swelling decreases due to the greater water absorption capacity of PEGMA-based systems compared to HEA-based systems for similar degrees of crosslinking [32,33,34].

In a basic medium, much higher and faster swelling is observed than in PBS. In this case, an increase in the amount of PEGDMA in the photopolymerizable mixture produces parts with lower swelling capacity. Since *β*-aminoesters are sensitive to the pH, that is, they are hydrolyzed much faster at a basic pH, the behavior of the samples represented in Figure 3 is compatible with the idea that the samples in PBS correspond to the type B networks in Figure 1, that is, swollen networks that have not undergone HCL hydrolysis. On the other hand, the samples in a basic medium correspond to the type C networks in Figure 1, because HCL hydrolysis gives rise to networks with a lower degree of crosslinking than type B networks, and therefore, with a greater swelling capacity. These C-type networks have, in principle, a crosslinking density, after HCL hydrolysis, defined by the amount of the SCL (see Table 1). This could explain why an increase in the amount of PEGDMA within the resin can produce parts with less pronounced swelling after hydrolysis.

Once the PEGDMA_7.5_-HEA_y_ series was studied, a fixed HEA/PEGMEA weight ratio was selected (60/25) and the PEGDMA/HCL weight ratio was varied (between 2/13 and 15/0) to analyze the role of the amount of HCL in the initial swelling and to determine whether the swelling of the partially hydrolyzed part can be modulated. The rationale behind the selection of the HEA/PEGMEA weight ratio of 60/25 was based on the fact that HEA-rich systems are less fragile, although this composition still contains a significant amount of hydrophilic PEGMEA macromonomer. This series has been labelled as PEGDMA_y_-HEA_60_, where *y* is the weight percentage of the PEGDMA. The characteristics of the samples studied in this second group are collected in Table 2. It can be seen that the molar crosslinking density (moles of PEGDMA + moles of HCL) increases with the amount of PEGDMA.

A rheological study was carried out again on three representative samples to address the printability of the prepared resins, as depicted in Table 2 (viscosity values for a shear rate of 1 s^−1^). All of them exhibited Newtonian behavior (see Figure S2 in SI) and a slight dependence on the composition, i.e., an increase in the amount of PEGDMA leads to a decrease in resin viscosity. However, the viscosities in all cases remain in the range of 0.012–0.024 Pa·s, suitable values for vat photopolymerization 3D printing [24,25].

**Figure 4:**
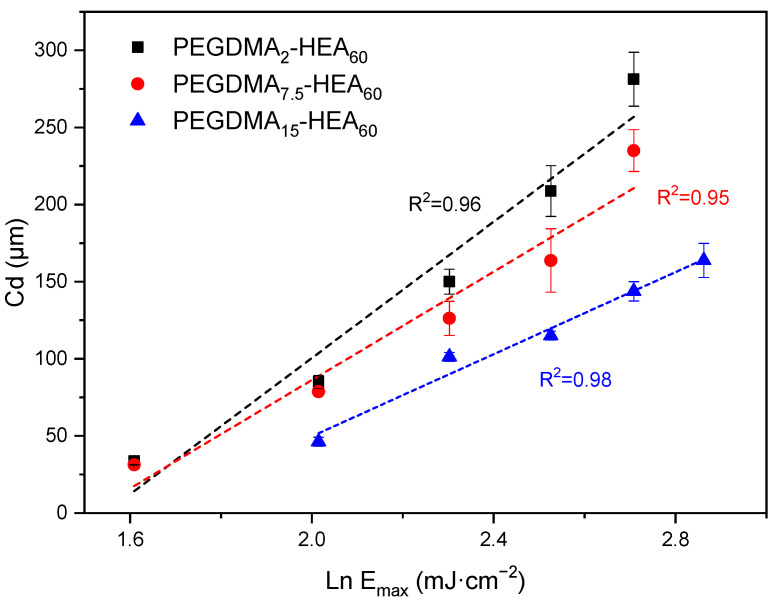
Jacobs working curves of the PEGDMA_y_-HEA_60_ series when exposed to 405 nm light (0.5 mW·cm^−2^). Data correspond to (from right to left in each group of data) exposure times of 35, 30, 25, 20, 15 and 10 s. Linear regressions are included.

From the Jacobs working curves (see Figure 4), the D_p_ and E_c_ values quoted in Table 2 were obtained. These results are in good agreement with those obtained with the PEGDMA_7.5_-HEA_y_ system, i.e., a higher nominal molar crosslinking density leads to greater curing depths. Again, a time of 20 s was found to be the most appropriate one to carry out the printing of all the pieces with common parameters.

**Figure 5:**
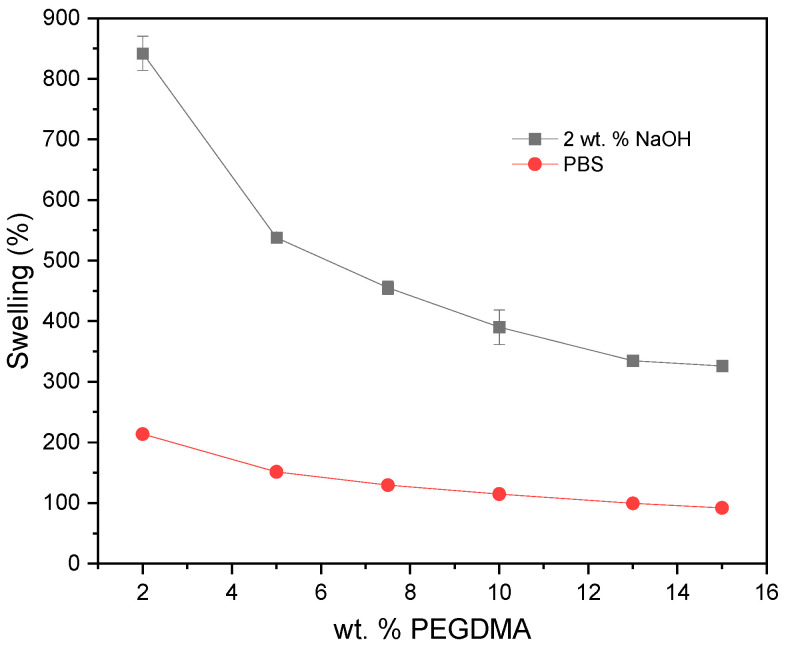
Red spheres: swelling at equilibrium in PBS. Black squares: swelling after 24 h in water with 2 wt. % NaOH; in both cases, the samples correspond to the PEGDMA_y_-HEA_60_ series. In the PBS data, the error bars are contained inside the symbols.

Pieces of 2.0 × 2.0 × 0.2 cm^3^ could be successfully printed using the conditions described in the Experimental Section. Following the procedure depicted above, these 3D printed pieces were immersed either in PBS or in basic water (2 wt. % of NaOH), and the swelling was determined via gravimetry as a function of the time (Figure 5). As occurred with the PEGDMA_7.5_-HEA_y_ series, these samples were found to reach equilibrium in PBS after a few days, with moderate swelling values in the range 100–200%. After 24 h in a basic medium, the swelling was, however, much higher and appeared to depend on the amount of PEGDMA. An increase in the amount of PEGDMA within the photopolymerizable resin resulted in 3D printed parts with lower swelling capacity. Again, the overall behavior in PBS and 2 wt. % NaOH was compatible with the re-adjustable hypothesis. In PBS, the hydrogel would correspond to a type B network (see Figure 1), whereas in the basic medium, there is already HCL hydrolysis and the networks are initially between B and C (when the hydrolysis of the HCL is not complete) and finally of type C. The dependence on the amount of PEGDMA is consistent with this process, because the swelling of the C type network is defined by the amount of SCL, that is, of PEGDMA. In the case of PBS, when there are B type networks, the global molar crosslinking density comprising the contribution of both stable and hydrolysable crosslinkers (SCL + HCL) increases only slightly with an increasing amount of PEGDMA, which explains the small swelling differences in PBS. The results of high swelling after treatment with an alkaline medium for networks with less PEGDMA are consistent with the literature data for similar HEA networks prepared with a very low crosslinking percentage [35].

**Figure 6:**
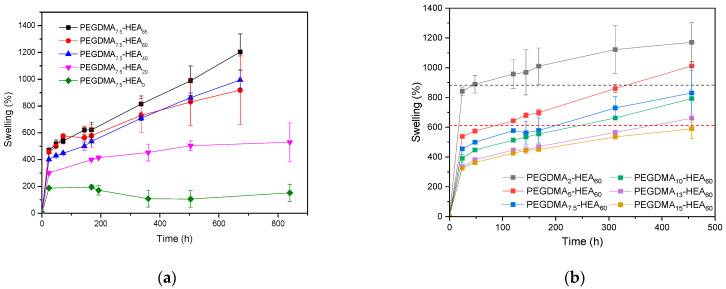
Swelling vs. time (from 1 h up to 20 days) in water with 2 wt. % NaOH for the PEGDMA_7.5_-HEA_y_ (**a**) and PEGDMA_y_-HEA_60_ (**b**) series.

While it appears that after 24 h in basic media the swelling started to stabilize, longer studies revealed an unexpected behavior. All the samples for both series described above did not reach the swelling equilibrium in 2 wt. % NaOH (see Figure 6). In most of the cases, a continuous gradual increase in swelling values was observed.

**Figure 7:**
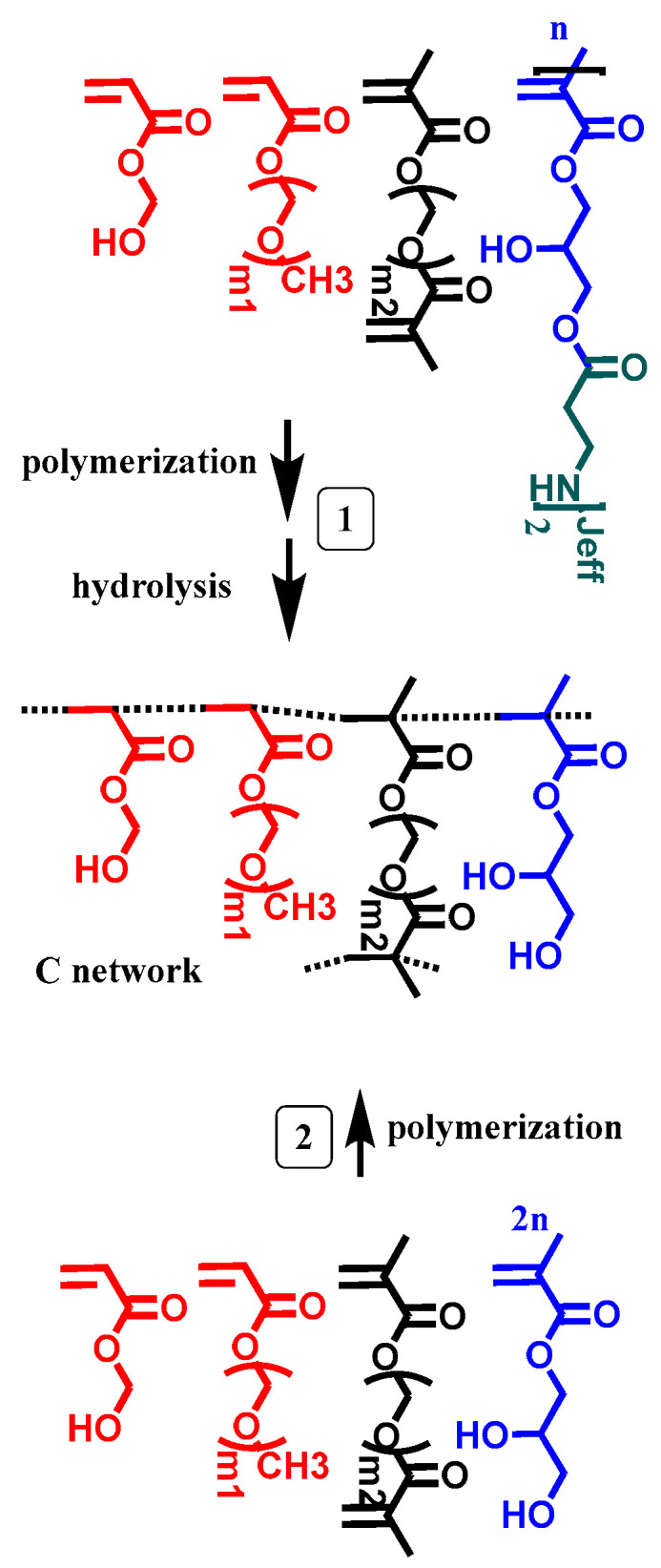
1: Scheme of the polymerization and hydrolysis of the PEGDMA_y_-HEA_60_ series. 2: Scheme of the preparation of reference structures carrying GMM. The jeff tag refers to the spacer of the Jeffamine (R of H_2_N-R-NH_2_).

Aiming to understand this behavior, reference structures were prepared from a formulation in which the HCL was replaced by twice the number of moles of the glycerol monomethacrylate, GMM (Figure 7). It is worth mentioning that GMM has the same structure as the HCL units upon hydrolysis in basic media, i.e., in the C network. By using these reference models, we expected to be able to determine a reference swelling for the C structure.

For this purpose, reference systems of the PEGDMA_2_-HEA_60_ and PEGDMA_5_-HEA_60_ samples were prepared (see the Experimental Section for examples of the system formulation and reference formulation). These reference networks were immersed in PBS until reaching equilibrium (using gravimetry), and these swellings in the equilibrium state are plotted in Figure 6b as dashed horizontal lines. These values are the values that a type C network should reach after breaking all the HCLs maintaining the 2 and 5 wt. % of PEGDMA. It can be seen how the PEGDMA_2_-HEA_60_ and PEGDMA_5_-HEA_60_ networks reach these reference data after about 50–100 h, although after that they continue to increase continuously.

The behavior at short times (up to 50 h) is in good agreement with the breakage of the majority of the HCL links occurring within the first 24 h. However, the fact that equilibrium is not reached must be explained by some other concomitant degradation process. In this sense, an NaOH solution may be aggressive enough to also break the acrylic side chain esters, even though they are not *β*-activated due to the presence of amines. There are also literature data evidencing that certain acrylates similar to those used in this work can be hydrolyzed in alkaline media [36]. To confirm if this process occurs in the proposed systems, a linear homopolymer of poly-HEA was synthesized, dissolved in D_2_O with a 2 wt. % NaOH, and monitored via ^1^H NMR. This analysis showed changes in the spectra that are compatible with the breakdown of the acrylates (see Figure S3 in SI). The broad peaks corresponding to the chain protons of pHEA (*CH_2_-CH*), initially centered at 2.4, 1.75, and 1.6 ppm, change shape and shift to broader peaks centered at 2.0 and 1.4 ppm, which is coincident with the chain displacements of polymethacrylic acid [37]. In addition, the signals of the side chain (*O-CH_2_-CH_2_-O* in HEA) also undergo changes compatible with hydrolysis, detecting the peaks corresponding to ethylene glycol at 3.5 ppm. Residual HEA peaks are observed centered at 4.2 and 4.0 ppms.

**Figure 8:**
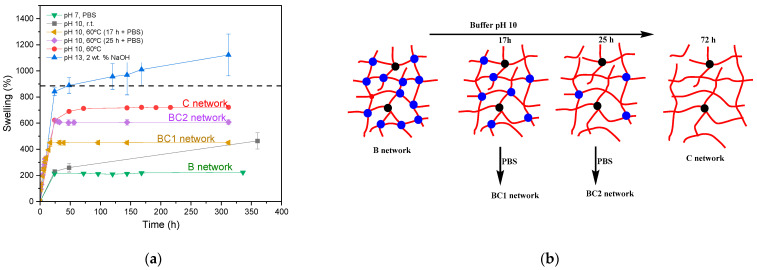
(**a**) Swelling of PEGDMA_2_-HEA_60_ in different media or temperatures. (**b**) Scheme of the hydrolytic process from a B to a C network. The blue and black knots represent, respectively, the knots corresponding to HCL and PEGDMA.

As the objective of this work was to demonstrate that it is possible to control the transition from a type B network to a type C network, the hydrolytic process of the HCL structures was addressed using less aggressive conditions that selectively allowed for the hydrolysis of the HCL. Specifically, a pH 10 buffer medium was employed, and the swelling variation was monitored both at room temperature (r.t., 20 °C) and at 60 °C to address if the temperature could play a role in the hydrolysis. For this study, the PEGDMA_2_-HEA_60_ sample has been chosen because it is the sample with the greatest readjustment capacity (remember that this formulation contains the largest amount of HCL, 13 wt. %). Figure 8 shows the variation in swelling of this system when submerged in pH 10 buffer at r.t. and at 60 °C, as well as in intermediate situations where the samples were first heated at 60 °C during either 17 h or 25 h and then transferred to PBS at r.t. The graph includes the data corresponding to the swelling change in an alkaline medium (2 wt. % NaOH) and in PBS as a function of the time. At pH 10 and 60 °C, a rapid increase in weight was observed until reaching a limit value close to 700% after about 70 h. The fact that an equilibrium limit value is reached, and that this value is slightly less than the swelling of the reference structure (dashed line), confirms that the pH 10 buffered medium is capable of hydrolyzing HCL but not the rest of the acrylic esters. At r.t., the swelling change is much slower, which means that the rate of hydrolysis of the HCL *β*-aminoesters in the pH 10 buffer can be controlled by the temperature.

Figure 8 shows that in PBS the equilibrium swelling value is reached after about 24 h and remains constant for weeks. This would be the swelling of the type B network. Networks with structures and swellings between network B and network C can be obtained via immersion in pH 10 for a selected time to allow partial hydrolysis of the *β*-aminoesters, followed by the stabilization of the structure via immersion in PBS, as shown in the diagram of Figure 8. This strategy was carried out via immersion in pH 10 for 17 and 25 h, followed by stabilization in PBS. These curves are represented in Figure 8 and show that it is possible to modulate the swelling between the two networks, B and C.

### 2.3. Proof of Concept of the Methodology: Preparation of 3D Printed Hydrogels with Tunable Swelling and Complex Structures

Once the re-adjustable behavior was thoroughly analyzed with square-shaped model parts, the dimensional readjustment in more complex pieces was addressed. Using the PEGDMA_2_-HEA_60_ formulation, spheres (SP) and tubes (T) with the dimensions indicated in Table 3 were prepared. In addition, a seahorse figure was printed to show this behavior in a piece with a more complex shape. The bottom image in Table 3 shows the freshly printed seahorse and when it was swollen after immersion in PBS for 72 h. It can be observed that the initial proportions are fully maintained, which indicates that the swelling was isotropic.

**Figure 9:**
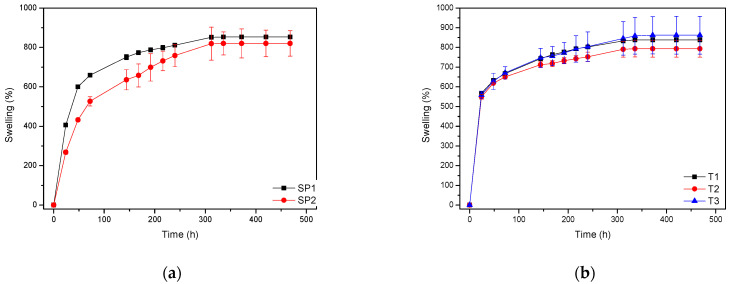
Swelling vs. time (from 1 h up to 20 days) of the spheres (**a**) and tubes (**b**) in pH 10 buffer at 60 °C.

Spheres SP1 and SP2 reached equilibrium swelling values after being immersed in PBS for 48 h, with values close to 150. In a basic medium, i.e., pH 10 buffer, they showed a continuous increase in swelling up to approximately 300 h, with the kinetics of the larger sphere being slower than those of the smaller sphere (see Figure 9a). The tubes displayed a similar behavior (Figure 9b). It must be taken into account that these pieces, especially the spheres, have larger dimensions than the pieces studied in Figure 8, which reached equilibrium within less than 100 h.

In any case, the printed parts in Table 3 show the same re-adjustability as previously described for the larger parts. In the case of the spheres, this capacity may be of interest in applications such as hydroponics and aquaponics crops [15], or in regenerative medicine to fill cavities [14], among others.

## 3. Conclusions

In this work, it has been shown that the strategy of using a mixture of stable (PEGDMA) and hydrolysable crosslinkers (HCL) allows for printing pieces via vat photopolymerization with different sizes and shapes, and with initial high crosslinking (PEGDMA and HCL knots) and then for transforming them into hydrogels via treatment in pH 10 buffer (with only remaining HCL knots). The swelling in water of the printed part ranges from the initial swelling (PEGDMA + HCL knots) to the swelling for total hydrolysis (only remaining PEGDMA knots are present). The intermediate swellings between these two states can be precisely controlled by adjusting the immersion time and temperature in pH 10 buffer. These target swellings have been stabilized by immersing the pieces in PBS at r.t.

## 4. Materials and Methods

### 4.1. Materials

The poly(ethylene glycol) methyl ether acrylate (PEGMEA480, MW 480), poly(ethylene glycol) dimethacrylate (PEGDMA550, Mw 550), sodium hydroxide (NaOH), 3-(acryloyloxy)-2-hydroxypropyl methacrylate, acetic acid, Sudan I, phenylbis(2,4,6-trimethyl benzoyl) phosphine oxide (BAPO), and phosphate-buffered saline (PBS, pH 7,4) were supplied by Merck and used as received without further purification.

The Jeffamine ED-600 (supplied by Huntsman) and glycerol monomethacrylate (GMM, supplied by Polysciences) were used as received.

2-hydroxy ethyl acrylate (HEA, supplied by TCI), azo-bis-isobutirenitrile (AIBN, supplied by Glentham) and dioxane (supplied by Panreac) were used as received.

The HCL was prepared as described previously from 3-(acryloyloxy)-2-hydroxypropyl methacrylate and Jeffamine using a catalytic quantity of acetic acid [20].

The synthesis of poly-HEA (pHEA) was carried out by thermal polymerization in solution. 665 mg of HEA were introduced into a vial, with a stirrer inside, and dissolved in 10 mL of dioxane. Next, 25 mg of AIBN were added. After that, the solution was left stirring for about 5–10 min for its complete homogenization and then nitrogen was bubbled for 5 min. Finally, the polymerization was allowed to proceed overnight in an oven at 60 °C. The next day, upon cooling to 5 °C separation into two phases was observed, which allows the dioxane to be easily removed.

### 4.2. Photopolymerizable Resin Formulations for 3D Printing

For each ink or formulation, a total of 20 g of precursor mixture was prepared. Examples of each type of system are detailed in Table 1 and Table 2. As an example of an ink for the PEGDMA_y_-HEA_60_ system, the formulation PEGDMA_5_-HEA_60_ was prepared by mixing 1.0 g of PEGDMA (1.8 mmol), 2.0 g of HCL (1.9 mmol), 5.0 g of PEGMEA (10.4 mmol) and 12.0 g of HEA (103.3 mmol). For this formulation, the reference formulation containing GMM instead of HCL was prepared by mixing 1.0 g of PEGDMA, 0.6 g of GMM (3.8 mmol), 5.0 g of PEGMEA and 12.0 g of HEA.

In all cases, 2.67 mg of Sudan I (1.35 wt. %) was added and the vessel was covered with aluminum foil. Then, 0.2 g (1 wt. %) of photoinitiator was added and the mixture was stirred for 5–10 min until it was completely homogeneous. The resin was then ready to be tested in the printer.

### 4.3. Selection of the 3D Printing and Post-Processing Parameters

The resin was added into the tank and the printing parameters chosen were as follows: layer 100 µm, raft height 1 mm, raft offset 4 mm, layer exposure time 20 s, exposure off time 5 s, bottom layer exposure time 60 s, bottom layers 5 pcs, platform lower speed 100 mm/m and 5 platform lift speed 100 mm/m. The printer used was the Zortrax Inkspire, using Z-Suite for Zortrax as the slicer program. After printing, the parts were washed in isopropanol for 5 min and post-cured for 30 min at room temperature in a UV light curing chamber.

### 4.4. Swelling Tests Performed on the 3D Printed Parts

The 3D printed pieces (2.0 × 2.0 × 0.2 cm^3^) were immersed in 15 mL of the corresponding media (PBS, buffer pH 10 or 2 wt. % NaOH in water) at room temperature or 60 °C. The pieces were weighted at different times to follow the swelling process. Swelling (S) was determined from Equation (1):

(1) S=(wS−wD)/wD

where wS and wD are the weights of the swollen and dry systems, respectively.

### 4.5. Methods

Rheological characterization of the uncured formulations was carried out at 25 °C using a strain-controlled rheometer (Waters, ARG2, TA Instruments), as detailed in the SI.

To obtain the Jacobs working curves [27,28,38], circular samples (diameter 2 cm) were cured with the Zortrax Inkspire 3D printer between the tank and a glass, with a constant intensity of 0.5 mW·cm^−2^ and varying the exposure times of between 10 and 35 s. Every sample was performed in duplicate. After the polymerization, the remaining solvent was extracted, and finally, the film was cleaned with paper. The curing depth (C_d_, μm) was obtained from the thickness of the samples, which was measured with a thickness gauge (Tactix 251420). The C_d_ is related to the irradiated light energy on the surface, E_max_, by Equation (2):

(2) $$C_{d}=D_{p}ln\left(\frac{E_{max}}{E_{c}}\right)$$

where E_max_ (mJ·cm^−2^) is given by the product of the exposure time and the energy of the lamp (0.5 mW·cm^−2^ in the Zortrax Inkspire 3D printer). E_c_ (mJ·cm^−2^) is the starting point for the transition from liquid to solid and D_p_ (μm) represents the penetration depth. Bearing in mind the properties of the 3D printed objects, it is necessary to obtain good adhesion between the layers. To achieve this, the use of layers with sufficient depth is essential, i.e., C_d_ > z, in our case Z = 100, because the stiffness of a polymer at the gel point (or below it) is too low to endure the printing process [28,39,40]. This expression equates to a linear line on a semilogarithmic plot of C_d_ on the *y*-axis versus E_max_ on the *x*-axis. The interception of the Jacobs working curve with the *x*-axis represents E_c_, whereas D_p_ is the slope of the linear line.

The hydrolysis of the poly-HEA ester was monitored by ^1^H NMR. 100 mg of polymer were dissolved in 0.6 mL of D_2_O at 2 wt. % of NaOH. This sample was monitored from time = 0 as indicated in Figure S3 in SI. ^1^H NMR spectra were recorded at room temperature on a Bruker Avance III HD-400 spectrometer ^(1^H 399.86 MHz) using D_2_O with 2 wt. % NaOH as solvent. Chemical shifts (d in ppm) are given from internal solvent, D_2_O, 4.8 ppm.

## Figures and Tables

**Table 1:** Data concerning the PEGDMA_7.5_-HEA_y_ series, including the penetration depth (D_p_) and critical exposure (E_c_) calculated from the Jacobs working curves.

Sample Label	PEGDMA/HCL/HEA/PEGMEA wt. %	PEGDMA/HCL/HEA/PEGMEA mol %	Crosslinker mol% (PEGDMA + HCL)	Viscosity (Pa·s) for a Shear Rate of 1 s^−1^	D_p_ (µm)	E_c_ (mJ·cm^−2^)
PEGDMA_7.5_-HEA_85_	7.5/7.5/85/0	1.8/1.0/97.2/0	2.8	0.012 ± 0.001	311	4.89
PEGDMA_7.5_-HEA_60_	7.5/7.5/60/25	2.3/1.2/87.7/8.8	3.5	0.019 ± 0.001	221	4.69
PEGDMA_7.5_-HEA_40_	7.5/7.5/40/45	3.0/1.6/75.0/20.4	4.6	0.026 ± 0.001	165	4.35
PEGDMA_7.5_-HEA_20_	7.5/7.5/20/65	4.2/2.2/52.4/41.2	6.4	0.034 ± 0.001	105	3.49
PEGDMA_7.5_-HEA_0_	7.5/7.5/0/85	6.9/3.6/0/89.5	10.5	0.041 ± 0.001	102	3.82

**Table 2:** Data concerning the PEGDMA_y_-HEA_60_ series, including the penetration depth (D_p_) and critical exposure (E_c_) calculated from the Jacobs working curves.

Sample Label	PEGDMA/HCL/HEA/PEGMEA wt. %	PEGDMA/HCL mol %	Viscosity (Pa·s) for a Shear Rate of 1 s^−1^	D_p_ (µm)	E_c_ (mJ·cm^−2^)
PEGDMA_2_-HEA_60_	2/13/60/25	0.6/2.1	0.024 ± 0.001	221	4.69
PEGDMA_5_-HEA_60_	5/10/60/25	1.5/1.6			
PEGDMA_7.5_-HEA_60_	7.5/7.5/60/25	2.3/1.2	0.019 ± 0.001	176	4.52
PEGDMA_10_-HEA_60_	10/5/60/25	3.1/0.8			
PEGDMA_13_-HEA_60_	13/2/60/25	4.0/0.3			
PEGDMA_15_-HEA_60_	15/0/60/25	4.6/0	0.012 ± 0.001	133	5.08

**Table 3:** Data concerning spheres, tubes and a seahorse printed from the formulation PEGDMA_2_-HEA_60_.

Spheres	Fresh printed parts, diameter (cm)		
	SP1: 0.50	SP2: 0.75	
	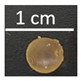 SP1	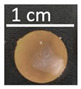 SP2	
	PBS (after 48 h), swelling		
	SP1 140 ± 1	SP2 142 ± 2	
	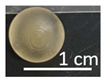 SP1	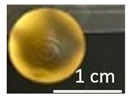 SP2	
	pH 10, 60 °C (after 372 h), swelling		
	SP1 853 ± 10	SP2 820 ± 73	
	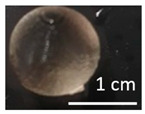 SP1	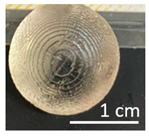 SP2	
Tubes	Fresh printed parts: diameter, wall thickness, length (mm)		
	T1: 10, 0.5, 10	T2: 15, 0.75, 15	T3: 20, 1, 20
	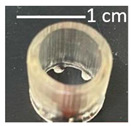 T1	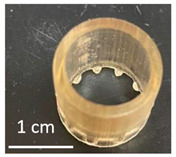 T2	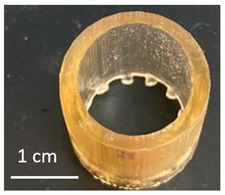 T3
	pH 10, 60 °C (after 144 h); swelling		
	T1 763 ± 9	T2 713 ± 15	T3 748 ± 48
Seahorse	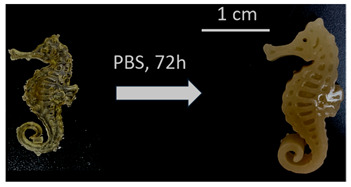		

## Data Availability

Non-confidential data will be published in digital.csic.

## Supplementary Materials

The following supporting information can be downloaded at https://www.mdpi.com/article/10.3390/gels9080600/s1, Figure S1: Viscosity values as a function of the shear rate for the PEGDMA_7.5_-HEA_y_ system; Figure S2: Viscosity values as a function of the shear rate for the PEGDMA_y_-HEA_60_ system; Figure S3: ^1^H NMR spectra of pHEA in D_2_0 with 2 wt. % of NaOH at time 0 (a) and 24 h (b).
