# Supplementary figures and images for: Vat Photopolymerization 3D Printing of Hydrogels with Re-Adjustable Swelling
Source: Gels. 2023 Jul 25;9(8):600. doi: 10.3390/gels9080600 (PMC10452991; doi:10.3390/gels9080600)

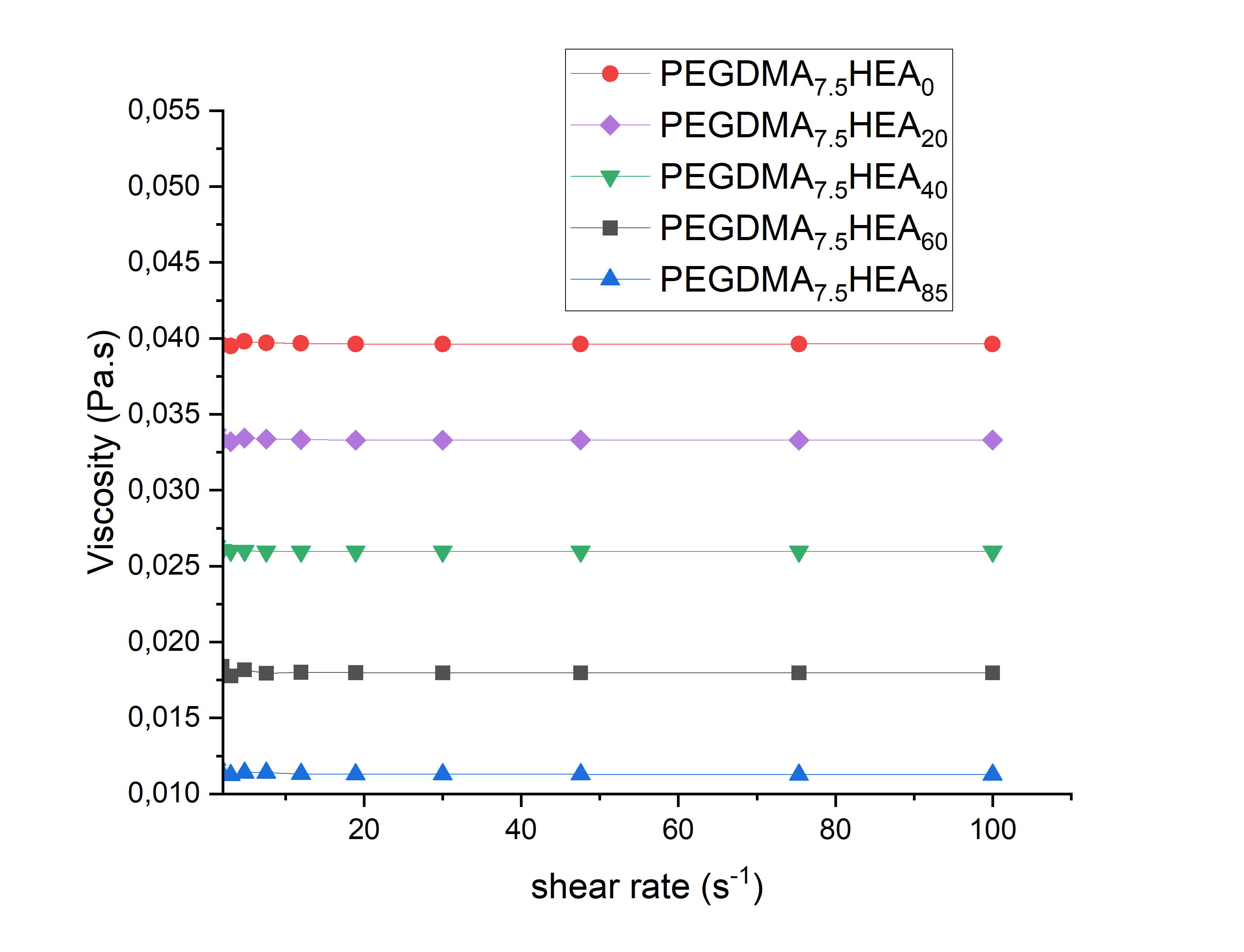

Supplement: Supplementary file 1 [file gels-09-00600-s001.zip › FigureS1.jpg]

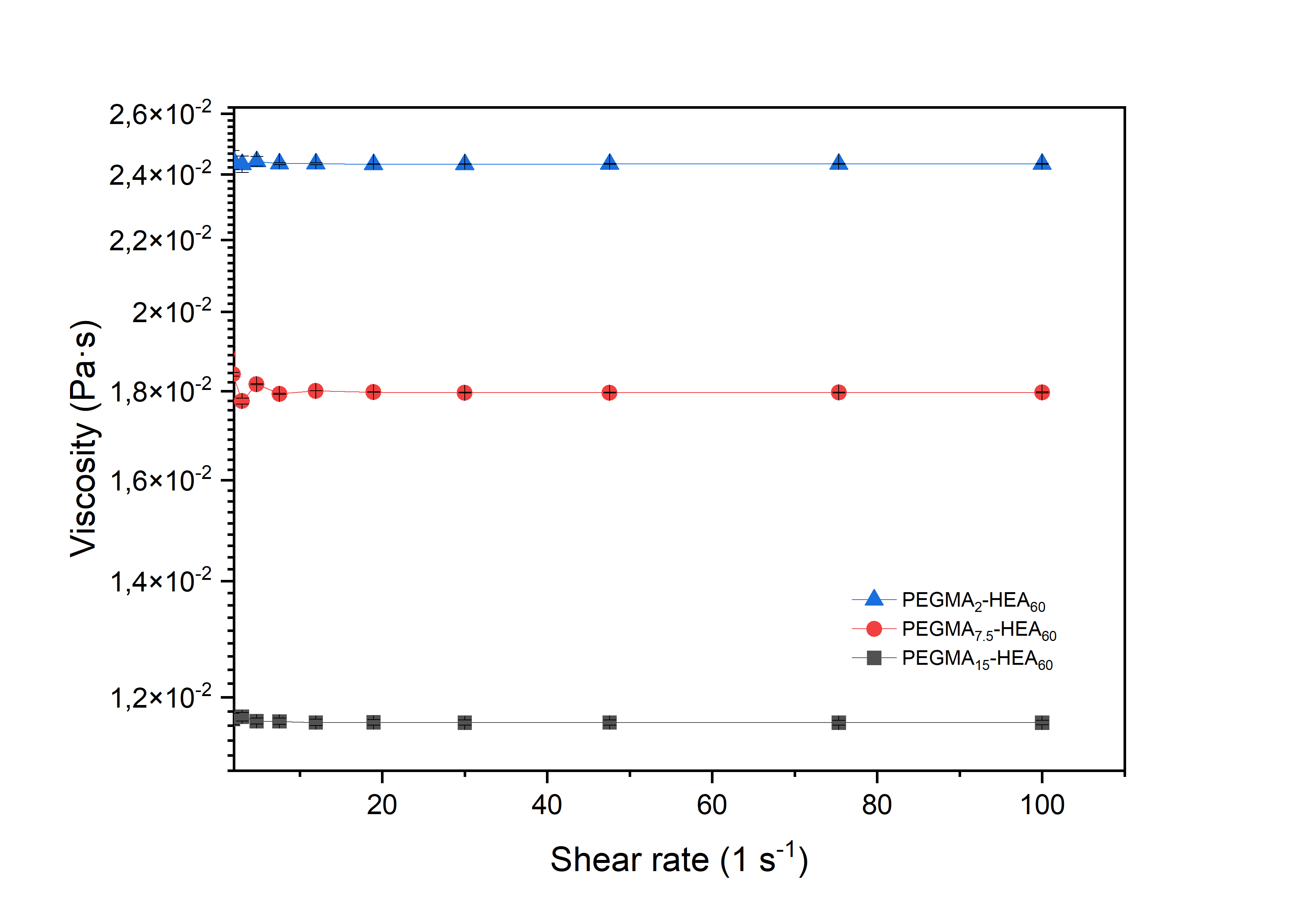

Supplement: Supplementary file 1 [file gels-09-00600-s001.zip › FigureS2.jpg]

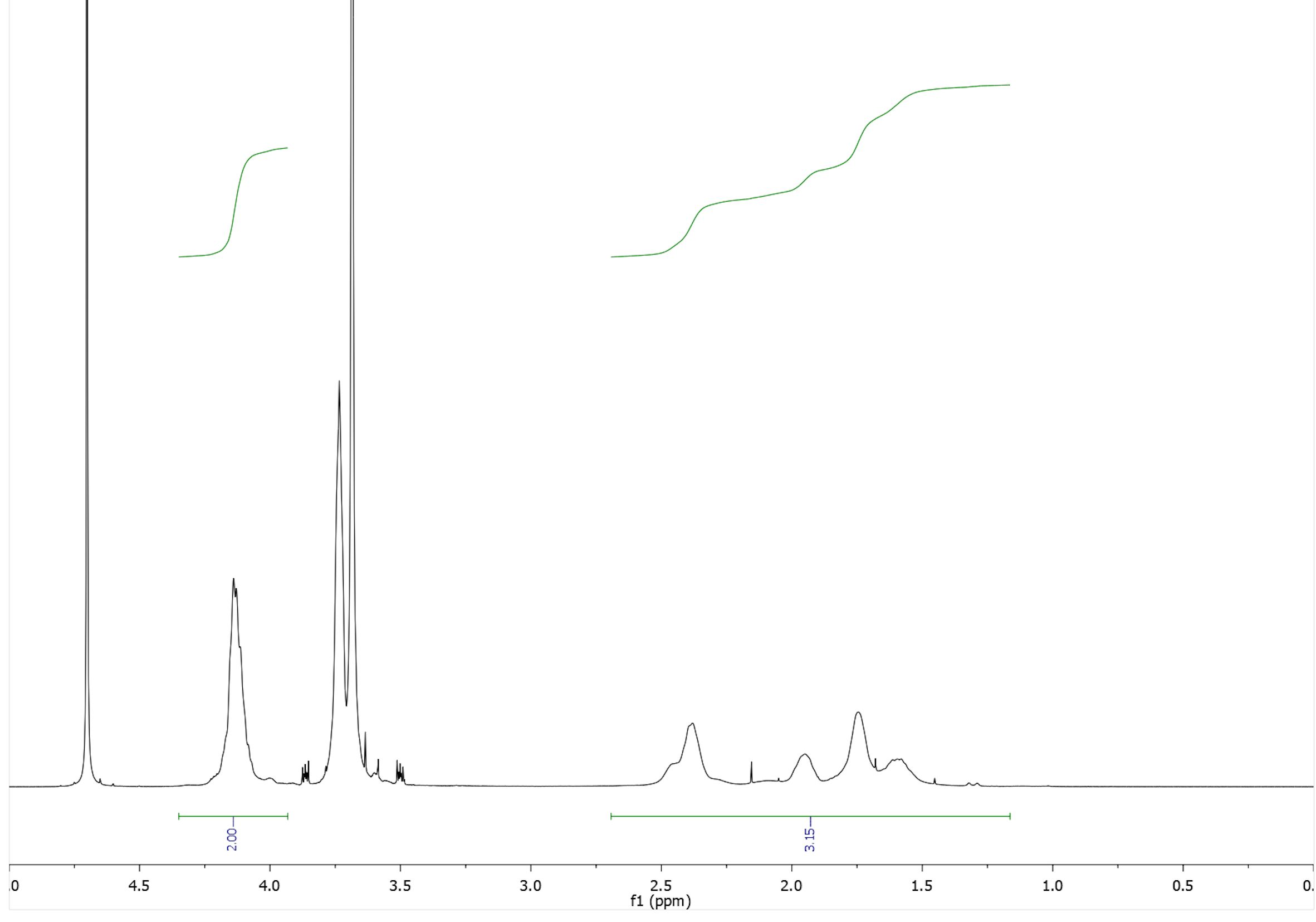

Supplement: Supplementary file 1 [file gels-09-00600-s001.zip › FigureS3a.jpg]

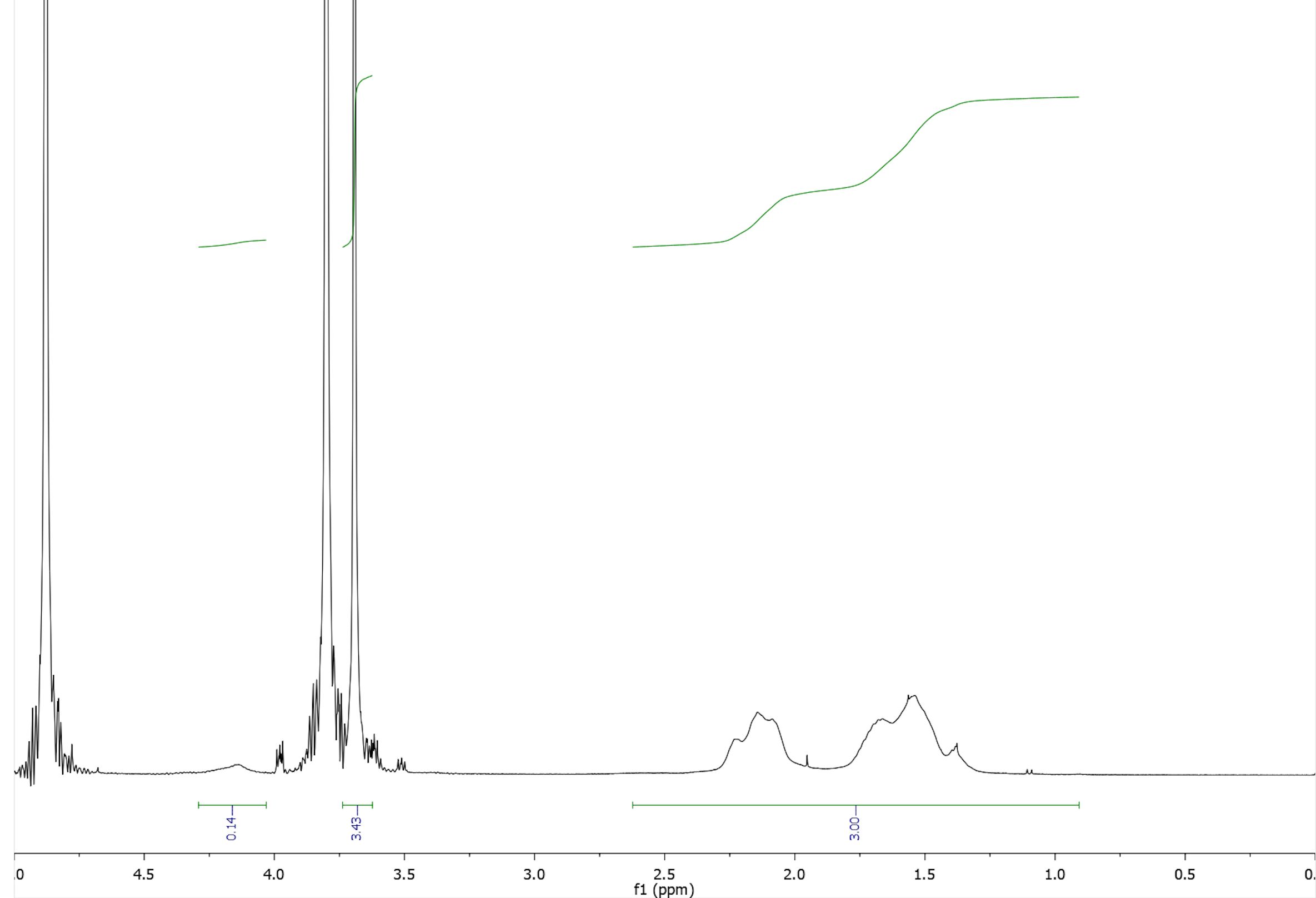

Supplement: Supplementary file 1 [file gels-09-00600-s001.zip › FigureS3b.jpg]
